# Digitalis use and risk of gastrointestinal cancers: A nationwide population-based cohort study

**DOI:** 10.18632/oncotarget.16151

**Published:** 2017-03-13

**Authors:** Shao-Hua Xie, Tomas Jernberg, Fredrik Mattsson, Jesper Lagergren

**Affiliations:** ^1^ Upper Gastrointestinal Surgery, Department of Molecular medicine and Surgery, Karolinska Institutet, Karolinska University Hospital, Stockholm, Sweden; ^2^ Department of Medical Epidemiology and Biostatistics, Karolinska Institutet, Stockholm, Sweden; ^3^ Department of Cardiology, Karolinska University Hospital, Stockholm, Sweden; ^4^ Division of Cancer Studies, King's College London, London, United Kingdom

**Keywords:** digitalis, digoxin, gastrointestinal cancers, medication, chemoprevention

## Abstract

**Background:**

Gastrointestinal cancers are characterized by a male predominance, suggesting a role of sex hormones. We hypothesized that digitalis medication, due to its estrogenic properties, decreases the risk of male-predominated gastrointestinal cancers.

**Results:**

Long -term digitalis use (≥2 years) was followed by decreased risk for several gastrointestinal cancers, but associations were statistically significant only for liver cancer (hazard ratio [HR]=0.40, 95% confidence interval (CI) 0.16-0.98). Short-term (<1 year) use was associated with an increased risk of esophageal squamous cell carcinoma (HR=1.79, 95% CI 1.01-3.17), colorectal cancer (HR=1.72, 95% CI 1.57-1.89), gallbladder cancer (HR=1.93, 95% CI 1.04-3.59), and pancreatic cancer (HR=1.33, 95% CI 1.00-1.76), but no such increase was found among long-term users.

**Methods:**

We performed a nationwide population-based cohort study in Sweden. Participants included 156,385 individuals using digitalis and a reference group of 551,933 users of organic nitrates between 2005 and 2013, who were identified in the Swedish Prescribed Drug Register. New diagnoses of gastrointestinal cancers were identified from the Swedish Cancer Register. Hazard ratios of gastrointestinal cancers in digitalis users compared to users of organic nitrates were calculated from Cox proportional hazards regression with adjustment for sex, age, municipality of residence and comorbidity.

**Conclusions:**

This study suggests a decreased risk of male-predominated gastrointestinal cancers, particularly of liver cancer, in long-term users of digitalis. Short-term use may be associated with an increased risk of esophageal squamous cell carcinoma, colorectal cancer, gallbladder cancer, and pancreatic cancer. The use of digitalis as preventive or therapeutic agents remains to be fully evaluated.

## INTRODUCTION

Gastrointestinal cancers account for approximately 30% of all cancers worldwide. Most of them are characterized by a remarkable male predominance in incidence [1, 2]. Esophageal adenocarcinoma has the highest male to female incidence ratio (up to 9:1), which is not fully explained by known risk factors [1, 3]. Sex hormones and reproductive factors, particularly a protective role of estrogenic exposures, have been hypothesized to be involved in the development of some male-predominated gastrointestinal cancers, but the existing evidence is inconclusive [1, 4–6].

Digoxin and other digitalis medications are widely used in treating common cardiovascular diseases, i.e. heart failure and atrial fibrillation. Interestingly, digitalis resembles estrogen chemically and can bind to estrogen receptors and exert estrogenic properties [7]. Recent pharmaco-toxicological studies suggest an anti-cancer potential of digoxin *in vitro*, and anti-cancer effects are being investigated in early-phase clinical cancer trials [8–11]. If sex hormones play a role in the etiology of gastrointestinal cancers, digitalis use may influence the risk of these cancers. However, this hypothesis has rarely been tested.

We hypothesized that digitalis use is associated with a reduced risk of gastrointestinal cancers, and that the magnitude of the association varies across anatomical sites and histological types of these malignancies, depending on the level of hormone-dependence. To test this hypothesis, we examined the association between digitalis use and the risk of gastrointestinal cancers in a nationwide population-based cohort study in Sweden.

## RESULTS

### Participants

The cohort of digitalis users consisted of 156,385 individuals (and 644,703 person-years). Of these, 83,925 (54%) were prevalent digitalis users and 72,460 (46%) were incident users. Digoxin was the predominant digitalis medication, accounting for over 99% of all total defined daily doses (DDDs) of prescribed digitalis, while the rest were all from digitoxin. The reference cohort of users of organic nitrates included 551,900 individuals (and 2,653,833 person-years). Some characteristics of the study participants using digitalis and organic nitrates only are presented in Table 1. There were fewer male digitalis users (46%) than male users of organic nitrates (53%). The mean age at first known exposure was 77.5 years in users of digitalis and 70.7 years in users of organic nitrates.

**Table 1:** Characteristics of study participants

	Users of digitalis		Users of organic nitrates	
	*N* (%)	Person-years	*N* (%)	Person-years
Sex				
Male	72,163 (46)	304,903	290,958 (53)	1,387,470
Female	84,222 (54)	339,799	260,975 (47)	1,266,363
Age at first known exposure, years				
<60	9,752 (6)	50,510	105,279 (19)	536,927
60 ˜	22,806 (15)	110,741	139,242 (25)	722,927
70 ˜	45,148 (29)	205,874	153,131 (28)	777,582
≥80	78,679 (50)	277,578	154,281 (28)	616,396
Duration of exposure				
Prevalent users	83,925 (54)	437,972	226,863 (41)	1,447,480
Incident users ^a^	72,460 (46)	206,731	325,070 (59)	1,206,353
<1 year	45,188 (29)	90,714	274,366 (50)	981,872
1 - 2 years	134,68 (9)	45,195	17,839 (3)	70,378
≥2 years	137,99 (9)	70,800	32,858 (6)	154,075
Total	156,385 (100)	644,703	551,933 (100)	2,653,833

^a^Five incident users had missing information on duration.

### Risk of gastrointestinal cancer

Both digitalis users and users of organic nitrates had elevated incidence rate ratios (IRRs) for most gastrointestinal cancers when compared to the general population (Table 2). When comparing digitalis users with users of organic nitrates, the IRRs were significantly increased for esophageal cancer (IRR=1.22, 95% confidence interval [CI] 1.01-1.46), colorectal cancer (IRR 1.23, 95% CI 1.17-1.29) and gallbladder cancer (IRR 1.63, 95% CI 1.19-2.25), but were closer to unity for most of the other cancers studied (Table 2).

**Table 2:** Risk of gastrointestinal cancers in users of digitalis and organic nitrates only using Poisson regression

Sites	Digitalis users (*N* = 156,385)		Users of nitrates only (*N* = 566,282)		IRR (95% CI) ^b^
	Cases (*N*)	IRR (95% CI) ^a^	Cases (*N*)	IRR (95% CI) ^a^	
Esophagus	156	1.39 (1.18, 1.63)	509	1.20 (1.09, 1.32)	1.22 (1.01, 1.46)
Stomach, carcia	73	1.30 (1.03, 1.65)	275	1.32 (1.16, 1.50)	1.04 (0.80, 1.36)
Stomach, non-cardia	227	1.08 (0.94, 1.23)	841	1.23 (1.14, 1.32)	0.90 (0.78, 1.05)
Small intestine	64	0.93 (0.72, 1.19)	255	0.97 (0.85,1.11)	1.00 (0.76, 1.33)
Colon or rectum	2,427	1.33 (1.28, 1.39)	7,166	1.14 (1.11, 1.17)	1.23 (1.17, 1.29)
Liver or intrahepatic bile duct	140	1.34 (1.13, 1.59)	539	1.24 (1.13, 1.36)	1.06 (0.88, 1.29)
Gallbladder	57	1.28 (0.98, 1.68)	131	0.85 (0.71, 1.22)	1.63 (1.19, 2.25)
Extrahepatic bile duct	27	1.03 (0.69, 1.51)	127	1.26 (1.05, 1.53)	NA
Ampullar of Vater	11	0.91 (0.49, 1.66)	62	1.35 (1.03, 1.77)	0.61 (0.32, 1.17)
Pancreas	271	1.18 (1.04, 1.33)	1,036	1.14 (1.07, 1.22)	1.08 (0.94, 1.24)

NA: not avaliable because the model did not converge.

^a^Incidence rate ratio (95% confidence interval) in individuals exposed to digitalis or nitrates compared with the general population, adjusted for sex, age, and calendar year.

^b^Incidence rate ratio (95% confidence interval) for digitalis users compared with users of organic nitrates, adjusted for sex, age, and calendar year.

The hazard ratios (HRs) from Cox regression in all digitalis users (Table 3) were similar to the IRRs from Poisson regression (Table 2). Digitalis use was associated with an increased HR for esophageal cancer (adjusted HR=1.26, 95% CI 1.05-1.51), colorectal cancer (HR 1.24, 95% CI 1.18-1.30), and gallbladder cancer (HR 1.66, 95% CI 1.20-2.28). Analyses by the duration of digitalis exposure in incident users indicated reduced HRs for several types of gastrointestinal cancers in participants who had used digitalis for at least 2 years, but was statistically significant only for liver cancer (HR 0.40, 95% CI 0.16-0.98). Short-term use (<1 year) of digitalis was associated with an increased HR for esophageal cancer (HR=1.46, 95% CI 1.01-2.11), colorectal cancer (HR 1.72, 95% CI 1.57-1.89), gallbladder cancer (HR=1.93, 95% CI 1.04-3.59) and pancreatic cancer (HR=1.33, 95% CI 1.00-1.76) (Table 3).

**Table 3:** Risk of gastrointestinal cancers in digitalis users compared with users of organic nitrates only by duration of exposure using Cox regression

Sites	Prevalent users		Incident users						All	
			< 1 year		1-2 years		≥ 2years			
	*N*	HR (95% CI) *	*N*	HR (95% CI) *	*N*	HR (95% CI) *	*N*	HR (95% CI) *	*N*	HR (95% CI) *
Esophagus ^a^	104	1.36 (1.08, 1.71)	35	1.46 (1.01, 2.11)	13	1.36 (0.63, 2.94)	4	0.51 (0.17, 1.50)	156	1.26 (1.05, 1.51)
Stomach, cardia ^b^	53	1.10 (0.80, 1.51)	14	1.21 (0.69, 2.15)	3	0.74 (0.16, 3.29)	3	0.85 (0.22, 3.27)	73	1.04 (0.80, 1.35)
Stomach, non-cardia ^c^	156	0.96 (0.80, 1.15)	46	1.05 (0.77, 1.44)	16	1.23 (0.61, 2.47)	9	0.68 (0.32, 1.43)	227	0.92 (0.79, 1.07)
Small intestine ^d^	47	1.06 (0.76, 1.48)	15	1.59 (0.90, 2.79)	2	0.29 (0.06, 1.48)	0	-	64	1.01 (0.77, 1.34)
Colon or rectum ^e^	1,578	1.24 (1.16, 1.31)	576	1.72 (1.57, 1.89)	145	1.33 (1.04, 1.69)	12	0.97 (0.78, 1.20)	2,427	1.24 (1.18, 1.30)
Liver or intrahepatic bile duct ^f^	96	1.02 (0.81, 1.29)	28	1.16 (0.77, 1.75)	10	0.90 (0.39, 2.10)	6	0.40 (0.16, 0.98)	140	0.94 (0.78, 1.14)
Gallbladder ^g^	36	1.76 (1.16, 2.68)	13	1.93 (1.04, 3.59)	6	3.85 (0.72, 20.55)	2	1.00 (0.18, 5.67)	57	1.66 (1.20, 2.28)
Extrahepatic bile duct ^g^	19	0.84 (0.50, 1.40)	5	1.04 (0.41, 2.67)	3	2.63 (0.38, 18.02)	0	-	27	0.82 (0.54, 1.26)
Ampullar of Vater ^g^	8	0.58 (0.27, 1.24)	2	0.92 (0.21, 4.11)	1	1.19 (0.07, 19.72)	0	-	11	0.63 (0.33, 1.20)
Pancreas ^h^	176	1.09 (0.92, 1.30)	59	1.33 (1.00, 1.76)	22	1.44 (0.79, 2.60)	14	0.90 (0.48, 1.69)	271	1.05 (0.92, 1.20)

*Hazard ratio (95% confidence interval), adjusted for sex, age, residence, and comorbidity.

^a^Adjusted comorbidities included obesity, gastroesophageal reflux disease, chronic obstructive pulmonary disease, chronic liver disease or liver cirrhosis, and diabetes.

^b^Adjusted comorbidities included obesity, gastroesophageal reflux disease, chronic obstructive pulmonary disease, and diabetes.

^c^Adjusted comorbidities included obesity, peptic ulcer, chronic obstructive pulmonary disease, chronic liver disease or liver cirrhosis, and diabetes.

^d^Adjusted comorbidities included obesity, Crohn's disease, Coeliac disease, chronic obstructive pulmonary disease, chronic liver disease or liver cirrhosis, and diabetes.

^e^Adjusted comorbidities included obesity, Crohn's disease, chronic obstructive pulmonary disease, chronic liver disease or liver cirrhosis, and diabetes.

^f^Adjusted comorbidities included obesity, viral hepatitis, chronic liver diseases or liver cirrhosis, chronic obstructive pulmonary disease, and diabetes.

^g^Adjusted comorbidities included obesity, cholelithiasis, viral hepatitis, chronic obstructive pulmonary disease, chronic liver disease or liver cirrhosis, and diabetes.

^h^Adjusted comorbidities included obesity, viral hepatitis, pancreatitis, cholelithiasis, chronic obstructive pulmonary disease, chronic liver disease or liver cirrhosis, and diabetes.

In analyses by histological type, an increased risk of esophageal cancer in digitalis users was found for squamous cell carcinoma (HR 1.61, 95% CI 1.22-2.13), but only restricted in short-term users when analyzed by duration of exposure (HR=1.79, 95% CI 1.01-3.17) (Table 4). Digitalis use was associated with a reduced but statistically insignificant HR for hepatocellular carcinoma regardless of the duration of exposure, and there was seemingly a trend toward lower HRs with longer duration of exposure. Use of digitalis for 2 years or more was associated with a possibly reduced HRs for esophageal squamous cell carcinoma, esophageal adenocarcinoma and cholangiocarcinoma, but without statistical significance (Table 4).

**Table 4:** Risk of selected gastrointestinal cancers in digitalis users compared with users of organic nitrates only by histologic type and by duration of exposure using Cox regression

Types of cancer	Prevalent users		Incident users						All	
			< 1 year		1-2 years		≥ 2years			
	*N*	HR (95% CI) *	*N*	HR (95% CI) *	*N*	HR (95% CI) *	*N*	HR (95% CI) *	*N*	HR (95% CI) *
Esophageal squamous cell carcinoma ^a^	48	1.70 (1.19, 2.40)	15	1.79 (1.01, 3.17)	8	2.39 (0.74, 7.73)	2	0.52 (0.11, 2.49)	73	1.61 (1.22, 2.13)
Esophageal adenocarcinoma ^b^	49	1.13 (0.81, 1.56)	18	1.25 (0.76, 2.08)	4	0.72 (0.22, 2.38)	2	0.48 (0.11, 2.17)	69	1.01 (0.78, 1.31)
Adenocarcinoma of gastric cardia ^b^	51	1.20 (0.87, 1.67)	12	1.17 (0.63, 2.16)	3	0.74 (0.16, 3.29)	3	0.95 (0.24, 3.76)	142	1.10 (0.84, 1.45)
Adenocarcinoma of the esophagus or gastric cardia ^b^	100	1.16 (0.92, 1.47)	30	1.22 (0.83, 1.80)	7	0.71 (0.28, 1.78)	5	0.67 (0.25, 1.82)	142	1.05 (0.87, 1.27)
Hepatocellular carcinoma ^c^	59	0.87 (0.65, 1.17)	14	0.94 (0.54, 1.65)	7	0.80 (0.29, 2.19)	3	0.34 (0.10, 1.18)	83	0.82 (0.64, 1.04)
Intrahepatic cholangiocarcinoma ^d^	22	1.23 (0.75, 2.02)	8	1.52 (0.71, 3.27)	2	1.09 (0.14, 8.77)	2	0.53 (0.11, 2.58)	34	1.12 (0.76, 1.65)
Extrahepatic cholangiocarcinoma ^d^	14	0.81 (0.45, 1.48)	5	1.22 (0.47, 3.15)	2	1.94 (0.24, 15.63)	0	-	21	0.80 (0.49, 1.29)
Cholangiocarcinoma of the liver or bile ducts ^d^	36	1.03 (0.70, 1.51)	13	1.38 (0.76, 2.51)	4	1.46 (0.33, 6.36)	2	0.33 (0.07, 1.51)	55	0.97 (0.72, 1.31)

*Hazard ratio (95% confidence interval), adjusted for sex, age, residence, and comorbidity.

^a^Adjusted comorbidities included chronic obstructive pulmonary disease, chronic liver disease or liver cirrhosis, and diabetes.

^b^Adjusted comorbidities included obesity, gastroesophageal reflux disease, chronic obstructive pulmonary disease, and diabetes.

^c^Adjusted comorbidities included obesity, viral hepatitis, chronic liver disease or liver cirrhosis, chronic obstructive pulmonary disease, and diabetes.

^d^Adjusted comorbidities included obesity, cholelithiasis, viral hepatitis, Crohn's disease, chronic liver disease or liver cirrhosis, chronic obstructive pulmonary disease, and diabetes.

**Figure 1:**
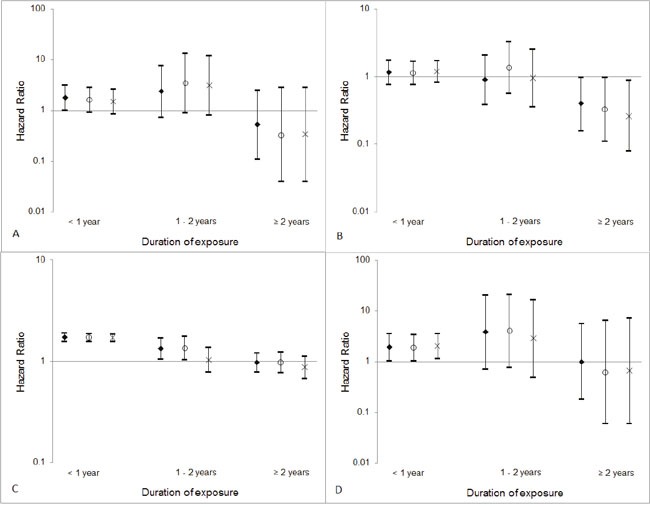
Hazard ratios and 95% confidence intervals for **(A)** esophageal squamous cell carcinoma, **(B)** liver cancer, **(C)** colorectal cancer, and **(D)** gallbladder cancer according to duration of exposure to digitalis and restriction period (diamonds: without restriction period; circles: excluding prescription within 6 months before cancer diagnosis; crosses: excluding prescription within 1year before cancer diagnosis).

The inclusion of a restriction period of 6 months or 1 year did not substantially alter the associations between the duration of exposure to digitalis and the risk of esophageal squamous cell carcinoma, liver cancer, colorectal cancer, or gallbladder cancer (Figure 1).

## DISCUSSION

This study found some support for the hypothesis that digitalis use for at least 2 years is associated with a decreased risk of several types of gastrointestinal cancer, although the results were statistically significant only for liver cancer. Shorter term use (<2 years) of digitalis was rather associated with an increased risk of esophageal squamous cell carcinoma, colorectal cancer, gallbladder cancer, and pancreatic cancer.

Strengths of this study include the large sample size, population-based cohort design, valid data on exposures and outcomes, complete follow-up of all participants and the evaluation of confounding by adjustment for potential confounders, including demographic characteristics and comorbidities, and by using a reference cohort exposure to organic nitrates. The use of a reference cohort and the sensitivity analyses with restriction periods reduced the risk of detection bias arising from more medical attention being given to patients with cardiovascular diseases. Despite the large sample size, a major limitation of the study is the low statistical power to assess long-term use of digitalis, which is likely the most important exposure from a cancer etiology perspective. Despite strongly reduced point estimates, most failed to reach the level of statistical significance. We were unable to assess the sex-specific effects of digitalis use due to the limited number of cancer patients, particularly the small number of female patients. Moreover, considering the large number of comparisons made in this report, the possibility of false discoveries by chance due to multiple testing could not be ruled out. Another limitation is that we were not able to adjust for all potential confounders, introducing a risk of residual confounding from unmeasured risk factors of gastrointestinal cancers. There were more patients of chronic obstructive pulmonary disease and diabetes in digitalis users compared with users of nitrates (Supplementary Table 1), indicating possible confounding from risk factors shared by gastrointestinal cancers and these conditions, e.g., tobacco smoking and dietary factors. Such possible residual confounding, although partially controlled for, might have contributed to the observed increased risk of esophageal squamous cell carcinoma, colorectal cancer, and pancreatic cancer in digitalis users and diluted expected inverse associations between digitalis exposure and the risk of other gastrointestinal cancers. A further weakness is that information on digitalis use was not available until the start of the study date (July 1, 2005), which introduced a risk of exposure misclassification. Any such misclassification is likely to be non-differential and should thus have attenuated the observed associations rather than explained them. Finally, this study was based on a relatively older Swedish population, and thus, our findings may not be representative of other populations or ethnicities.

Recent studies have revealed an increased risk of cancers of the breast and uterus, and a decreased risk of prostate cancer following digitalis use [12–17]. Our study is the first to investigate digitalis use in relation to the risk of gastrointestinal cancers other than colorectal cancer. A case-control study from the United Kingdom found an increased risk of colorectal cancer among users of digitalis compared with non-users [18], a finding supported by our study. These findings contradict the hypothesis of a protective effect as suggested by previous epidemiological findings of an inverse association between estrogens and colorectal cancer risk in postmenopausal women [6, 19, 20], although increased risk of colorectal cancer associated with estrogenic exposures has been reported in some other studies [21, 22]. The possibly increased risk of esophageal squamous cell carcinoma, colorectal cancer, gallbladder cancer and pancreatic cancer among digitalis users in the present study needs confirmation in other studies. Residual confounding by e.g. tobacco smoking cannot be excluded, particularly since no association was found among long-term users of digitalis.

The possibly decreased risk of esophageal squamous cell carcinoma, esophageal adenocarcinoma, gastric cancer, and liver cancer in long-term users of digitalis is interesting and supports the estrogen hypothesis of the study. An anti-cancer potential of digitalis has been found in pharmaco-toxicological studies *in vitro* [8, 10, 11]. The possible biological mechanisms include, but are not limited to, inhibition of proliferation in cancer cells, controlling of the cell cycle, induction of apoptosis, and anti-inflammatory properties [8, 10, 11, 23]. Digitalis is a phytoestrogen that binds to the estrogen receptors through a lower affinity than for estrogen itself [7, 24]. The decreased risk associated with long-term exposure to digitalis for gastrointestinal cancers, particularly liver cancer, may be explained by a protective role of estrogenic exposures. This might contribute to the male predominance in some of these tumors. On the contrary, the increased risk of esophageal squamous cell carcinoma, colorectal cancer, gallbladder cancer, and pancreatic cancer may be related to non-estrogenic or pathways other than the aforementioned mechanisms, which merits further investigation. There is no strong reason to believe that estrogens are involved in the etiology of esophageal squamous cell carcinoma, colorectal cancer, gallbladder cancer, or pancreatic cancer, which have a weak-to-moderate male predominance explained by other risk factors or even a female predominance. It is well-established that the principal pharmacological effect of digitalis is mediated through the inhibition of Na^+^/K^+^ ATPase, leading to alterations of downstream transduction pathways [10, 11]. Particularly, alterations of intracellular calcium homeostasis following the inhibition of Na^+^/K^+^ ATPase may influence the regulation of proliferation, apoptosis, autophagy, and tumor differentiation, which may lead to an altered cancer risk [25, 26]. Given the complexity of the enzyme structure and the fact that its exact function and regulation in normal cells and cancer cells are largely unclear [10], the role of digitalis in cancer development and whether it is dependent on specific cellular contexts in given cancer tissues need to be further explored. Although assessment of the anti-cancer activity of digitalis has already reached early-phase clinical trials [10, 11], the use of digitalis as preventive or therapeutic agents remains to be fully evaluated.

In summary, this large population-based cohort study suggests that long-term use of digitalis is followed by a decreased risk of liver cancer, and possibly also of esophageal squamous cell carcinoma, esophageal adenocarcinoma, and gastric cancer. Short-term use of digitalis may increase the risk of esophageal squamous cell carcinoma, colorectal cancer, gallbladder cancer, and pancreatic cancer. However, the statistical power was limited by the rarity of these cancers, and these findings remain to be confirmed in other large-scale investigations with longer follow-up.

## MATERIALS AND METHODS

### Design

The source population consisted of all residents in Sweden from July 1, 2005 (when the Swedish Prescribed Drug Register started) to December 31, 2013 [27]. The cohort of digitalis users consisted of all individuals who had been dispensed any digitalis with the Anatomical Therapeutic Chemical (ATC) classification code “C01AA” according to the Swedish Prescribed Drug Register. To counteract possible information bias and confounding related to cardiovascular diseases and risk factors for such diseases, we compared the exposed cohort to a reference cohort of individuals who had been exposed to the angina pectoris drugs organic nitrates (ATC code C01DA), but had no digitalis medication. The 10-digit unique personal identity number assigned to all Swedish residents allowed us to obtain additional information through linkage of study participants to other nationwide registers. We used the Swedish Cancer Register to exclude individuals with any diagnosis of gastrointestinal cancers (codes according to the International Classification of Diseases, version 7 [ICD-7]: 150-157) before their first known exposure to digitalis or organic nitrates. Cohort members were followed up until the occurrence of any new gastrointestinal cancer, death (through linkage to the Swedish Causes of Death Register), or December 31, 2013 (end of the study), whichever occurred first. This study was approved by the Regional Ethical Review Board in Stockholm, Sweden (Protocol Number: 2015/3: 8).

### Exposures, outcomes and covariates

We extracted all records of digitalis and organic nitrates prescriptions dispensed during the study period from the Swedish Prescribed Drug Register. Obtained information included dates of prescription and dispensing, the dosage, and the amount of the prescribed drugs in terms of the numbers of DDDs per package and the number of dispensed packages.

New diagnoses of gastrointestinal cancers were identified through linkage to the Swedish Cancer Register, which has at least 96% coverage of all malignancies in the whole nation and 99% of reported cases were morphologically verified [28]. Potential confounding factors included in the analyses were age, sex, municipality of residence, and comorbidity. Information on comorbidities was obtained through linkage to the Swedish Patient Register. We restricted the comorbidities to diagnoses within 20 years before the end of follow-up and after 1 January 1987, from which the Patient Register achieved complete nationwide coverage [29]. Candidate comorbidities included known risk conditions for specific gastrointestinal cancers (e.g., gastroesophageal reflux disease for esophageal adenocarcinoma), chronic obstructive pulmonary disease, chronic liver disease or liver cirrhosis, and diabetes (Supplementary Table 1). The latter three groups of comorbidities were used as indirect indicators for tobacco smoking, heavy alcohol use, and both, respectively.

### Statistical analysis

To facilitate the possible comparison with other studies where when only tabulated data are available instead of individual records, we calculated the IRRs of gastrointestinal cancers separately for individuals exposed to digitalis and those exposed to organic nitrates. The IRRs were calculated by dividing the observed incidence rates by the baseline incidence rates in the corresponding population. We also computed the IRRs of these cancers by directly comparing digitalis users with users of organic nitrates. All IRRs and their 95% CIs were estimated by log-linear Poisson regression with adjustment for age (in 5-year groups), sex, and calendar year, where the count of cancer cases was the dependent variable with the natural logarithm of person-time as the offset term [30].

We also performed Cox proportion hazards regressions to estimate the HRs for gastrointestinal cancers in digitalis users compared to users of organic nitrates while adjusting for sex, age at first known exposure (<50 years, 50-59, 60-69, 70-79, and ≥ 80 years), municipality of residence, and cancer type-specific comorbidities. Chronic obstructive pulmonary disease was used as an indirect marker for tobacco smoking and other respirable environmental exposures, chronic liver disease or liver cirrhosis as for heavy alcohol use, and diabetes as for both. Considerations of other comorbidities to be included in each adjustment were based on of their association with the specific type of cancer. We further performed analyses stratified by histological type, i.e., separately for esophageal squamous cell carcinoma (ICD-7 codes: 150; histological codes using ICD for Oncology, version 3 [ICD-O-3]: 8050-8078, 8083-8084), adenocarcinoma of esophagus and gastric cardia (ICD-7 codes: 150, 151.1; histological codes: 8140-8141, 8143-8145, 8190-8231, 8260- 8263, 8310, 8401, 8480-8490, 8550-8551, 8570-8574, 8576), hepatocellular carcinoma (ICD-7 code: 155.0; histological code: 8170-8176), and cholangiocarcinoma (ICD-7 code: 155.0; histological codes: 8050, 5140-8141, 8160-8161, 8260, 8440, 8480-8500, 8570-8572; or ICD-7 codes: 155.1-155.9; histological codes: 8010, 8020, 8041, 8070, 8140, 8160, 8161, 8260, 8310, 8480, 8490, 8560, 8162-8163), given the known etiological heterogeneity in cancers of different histological type even in the same anatomical sites.

Since the dosage of digitalis was almost always standard with a narrow range within Sweden, we evaluated only potential dose-response associations in terms of duration. Because a prescription is valid only for up to 12 months in Sweden, we defined individuals with their first prescription in the first 12 months of the study as “prevalent users”, for whom information on exposure prior to the study was unclear. The remaining individuals were defined as “incident users”. The duration of exposure in incident users was estimated by the DDDs dispensed divided by 365.25 (days per year), and then categorized into three groups: <1 year, 1-2 years, and ≥2 years of duration. To assess potential detection bias from medical attention related to digitalis use, which might have led to an earlier diagnosis of cancer, we performed sensitivity analyses ignoring exposure within a restriction period of 6 months or 1 year before the cancer diagnosis was identified. All statistical analyses were performed using the statistical package SAS version 9.4 (SAS Institute Inc., Cary, NC, USA). A *p* value less than 0.05 was considered statistically significant.

## SUPPLEMENTARY MATERIALS FIGURES AND TABLES



## References

[1] Xie SH, Lagergren J (2016). The Male Predominance in Esophageal Adenocarcinoma. Clin Gastroenterol Hepatol.

[2] Ferlay J, Soerjomataram I, Ervik M, Dikshit R, Eser S, Mathers C, Rebelo M, Parkin DM, Forman D, Bray F ((2013)). GLOBOCAN 2012 v1.0, Cancer Incidence and Mortality Worldwide: IARC CancerBase No. 11.

[3] Freedman ND, Derakhshan MH, Abnet CC, Schatzkin A, Hollenbeck AR, McColl KE (2010). Male predominance of upper gastrointestinal adenocarcinoma cannot be explained by differences in tobacco smoking in men versus women. Eur J Cancer.

[4] Camargo MC, Goto Y, Zabaleta J, Morgan DR, Correa P, Rabkin CS (2012). Sex hormones, hormonal interventions, and gastric cancer risk: a meta-analysis. Cancer Epidemiol Biomarkers Prev.

[5] Cook MB, Dawsey SM, Freedman ND, Inskip PD, Wichner SM, Quraishi SM, Devesa SS, McGlynn KA (2009). Sex disparities in cancer incidence by period and age. Cancer Epidemiol Biomarkers Prev.

[6] Barzi A, Lenz AM, Labonte MJ, Lenz HJ (2013). Molecular pathways: Estrogen pathway in colorectal cancer. Clin Cancer Res.

[7] Rifka SM, Pita JC, Loriaux DL (1976). Mechanism of interaction of digitalis with estradiol binding sites in rat uteri. Endocrinology.

[8] Zhang H, Qian DZ, Tan YS, Lee K, Gao P, Ren YR, Rey S, Hammers H, Chang D, Pili R, Dang CV, Liu JO, Semenza GL (2008). Digoxin and other cardiac glycosides inhibit HIF-1alpha synthesis and block tumor growth. Proc Natl Acad Sci U S A.

[9] Kepp O, Menger L, Vacchelli E, Adjemian S, Martins I, Ma Y, Sukkurwala AQ, Michaud M, Galluzzi L, Zitvogel L, Kroemer G (2012). Anticancer activity of cardiac glycosides: At the frontier between cell-autonomous and immunological effects. Oncoimmunology.

[10] Alevizopoulos K, Calogeropoulou T, Lang F, Stournaras C (2014). Na+/K+ ATPase inhibitors in cancer. Curr Drug Targets.

[11] Slingerland M, Cerella C, Guchelaar HJ, Diederich M, Gelderblom H (2013). Cardiac glycosides in cancer therapy: from preclinical investigations towards clinical trials. Invest New Drugs.

[12] Biggar RJ, Wohlfahrt J, Oudin A, Hjuler T, Melbye M (2011). Digoxin use and the risk of breast cancer in women. J Clin Oncol.

[13] Biggar RJ, Wohlfahrt J, Melbye M (2012). Digoxin use and the risk of cancers of the corpus uteri, ovary and cervix. Int J Cancer.

[14] Kaapu KJ, Murtola TJ, Maattanen L, Talala K, Taari K, Tammela TL, Auvinen A (2016). Prostate cancer risk among users of digoxin and other antiarrhythmic drugs in the Finnish Prostate Cancer Screening Trial. Cancer Causes Control.

[15] Wright JL, Hansten PD, Stanford JL (2014). Is digoxin use for cardiovascular disease associated with risk of prostate cancer?. Prostate.

[16] Ahern TP, Tamimi RM, Rosner BA, Hankinson SE (2014). Digoxin use and risk of invasive breast cancer: evidence from the Nurses' Health Study and meta-analysis. Breast Cancer Res Treat.

[17] Platz EA, Yegnasubramanian S, Liu JO, Chong CR, Shim JS, Kenfield SA, Stampfer MJ, Willett WC, Giovannucci E, Nelson WG (2011). A novel two-stage, transdisciplinary study identifies digoxin as a possible drug for prostate cancer treatment. Cancer Discov.

[18] Boursi B, Haynes K, Mamtani R, Yang YX (2014). Digoxin use and the risk for colorectal cancer. Pharmacoepidemiol Drug Saf.

[19] Chlebowski RT, Wactawski-Wende J, Ritenbaugh C, Hubbell FA, Ascensao J, Rodabough RJ, Rosenberg CA, Taylor VM, Harris R, Chen C, Adams-Campbell LL, White E (2004). Women's Health Initiative I. Estrogen plus progestin and colorectal cancer in postmenopausal women. N Engl J Med.

[20] Murphy N, Strickler HD, Stanczyk FZ, Xue X, Wassertheil-Smoller S, Rohan TE, Ho GY, Anderson GL, Potter JD, Gunter MJ (2015). A Prospective Evaluation of Endogenous Sex Hormone Levels and Colorectal Cancer Risk in Postmenopausal Women. J Natl Cancer Inst.

[21] Zervoudakis A, Strickler HD, Park Y, Xue X, Hollenbeck A, Schatzkin A, Gunter MJ (2011). Reproductive history and risk of colorectal cancer in postmenopausal women. J Natl Cancer Inst.

[22] Gunter MJ, Hoover DR, Yu H, Wassertheil-Smoller S, Rohan TE, Manson JE, Howard BV, Wylie-Rosett J, Anderson GL, Ho GY, Kaplan RC, Li J, Xue X (2008). Insulin, insulin-like growth factor-I, endogenous estradiol, and risk of colorectal cancer in postmenopausal women. Cancer Res.

[23] Yang Q, Huang W, Jozwik C, Lin Y, Glasman M, Caohuy H, Srivastava M, Esposito D, Gillette W, Hartley J, Pollard HB (2005). Cardiac glycosides inhibit TNF-alpha/NF-kappaB signaling by blocking recruitment of TNF receptor-associated death domain to the TNF receptor. Proc Natl Acad Sci U S A.

[24] Biggar RJ (2012). Molecular pathways: digoxin use and estrogen-sensitive cancers—risks and possible therapeutic implications. Clin Cancer Res.

[25] Tesselaar MH, Crezee T, Swarts HG, Gerrits D, Boerman OC, Koenderink JB, Stunnenberg HG, Netea MG, Smit JW, Netea-Maier RT, Plantinga TS (2017). Digitalis-like Compounds Facilitate Non-Medullary Thyroid Cancer Redifferentiation through Intracellular Ca2+, FOS, and Autophagy-Dependent Pathways. Mol Cancer Ther.

[26] Farfariello V, Iamshanova O, Germain E, Fliniaux I, Prevarskaya N (2015). Calcium homeostasis in cancer: A focus on senescence. Biochim Biophys Acta.

[27] Wettermark B, Hammar N, Fored CM, Leimanis A, Otterblad Olausson P, Bergman U, Persson I, Sundstrom A, Westerholm B, Rosen M (2007). The new Swedish Prescribed Drug Register—opportunities for pharmacoepidemiological research and experience from the first six months. Pharmacoepidemiol Drug Saf.

[28] Barlow L, Westergren K, Holmberg L, Talback M (2009). The completeness of the Swedish Cancer Register: a sample survey for year 1998. Acta Oncol.

[29] Ludvigsson JF, Andersson E, Ekbom A, Feychting M, Kim JL, Reuterwall C, Heurgren M, Olausson PO (2011). External review and validation of the Swedish national inpatient register. BMC Public Health.

[30] SAS Institute Inc. (2008). SAS/STAT 9.2 Use's Guide.

