# Supplementary material for: Digitalis use and risk of gastrointestinal cancers: A nationwide population-based cohort study
Source: Oncotarget. 2017 Mar 13;8(21):34727–35. doi: 10.18632/oncotarget.16151 (PMC5471006; doi:10.18632/oncotarget.16151)
Supplement: Supplementary file 1 [file oncotarget-08-34727-s001.pdf]

Digitalis use and risk of gastrointestinal cancers: A nationwide population-based cohort study

SUPPLEMENTARY TABLE

Supplementary Table 1: Distribution of comorbidities in digitalis users and users of organic nitrates only

	ICD-9-SE codes	ICD-10-SE codes	Users of digitalis Number (%)	Users of organic nitrates only Number (%)	*P* value [a]
Obesity [b]	278A	E66	5 536 (3.5)	22 567 (4.1)	< 0.001
Gastroesophageal reflux disease	787B, 530B, 530C, 553D	R12, K20, K21, K44	12 979 (8.3)	65 934 (12.0)	< 0.001
Peptic ulcer	531-533	K25-K27	10 489 (6.7)	30 785 (5.6)	< 0.001
Viral hepatitis	070	B15-B19	744 (0.5)	3 531 (0.6)	< 0.001
Chronic liver diseases and cirrhosis	571	K70, K71.7, K73, K74, K75.4	1 525 (1.0)	3 903 (0.7)	< 0.001
Cholelithiasis	574	K80	11 691 (7.5)	40 879 (7.4)	0.356
Pancreatitis	577A, 577B	K85, K86.0, K86.1	2 978 (1.9)	10 352 (1.9)	0.461
Chronic obstructive pulmonary disease	490, 491, 492, 494, 496	J40-J44, J47	22 954 (14.7)	54 561 (9.9)	< 0.001
Diabetes	250	E10-E14	38 286 (24.5)	114 399 (20.7)	< 0.001
Crohn's disease	555	K50	797 (0.5)	3 336 (0.6)	< 0.001
Coeliac disease	579A	K90.0	538 (0.3)	2 454 (0.4)	< 0.001

ICD-9-SE: International Classification of Diseases, 9th Swedish version; ICD-10-SE: International Classification of Diseases, 10th Swedish version.

[a] Chi-square test comparing digital users with users of nitrates.

[b] Obesity was underdiagnosed in the Swedish National Patient Register, but might indicate severer conditions.
